# Safety and Tolerability of bi-monthly Aripiprazole: findings from a real-world descriptive study

**DOI:** 10.1192/j.eurpsy.2026.10419

**Published:** 2026-07-31

**Authors:** T. Prodi, R. Leuzzi, M. C. Angeletti, K. La Monica, R. Anniverno, B. Dell’Osso, M. Olivola

**Affiliations:** 1 Department of Biomedical and Clinical Sciences “Luigi Sacco”, University of Milan; 2 Department of Mental Health and Addiction Services, ASST Fatebenefratelli-Sacco; 3 ”Aldo Ravelli” Center for Nanotechnology and Neurostimulation, University of Milan, Milan, Italy; 4 Department of Psychiatry and Behavioral Sciences, Stanford University, Stanford, United States; 5 Centro per lo studio dei meccanismi molecolari alla base delle patologie neuro-psico-geriatriche”, University of Milan, Milan; 6 Department of Brain and Behavioural Sciences, University of Pavia, Pavia, Italy

## Abstract

**Introduction:**

Non-adherence remains one of the major challenges in the treatment of severe and persistent mental disorders. Long-acting injectable (LAI) formulations of antipsychotics may improve adherence and reduce relapse rates. Aripiprazole LAI 960 mg every two months (Q2M) is a novel treatment option designed to simplify management and support continuity of care.

**Objectives:**

Through the present study, we aimed to evaluate the efficacy, safety, and tolerability of bi-monthly aripiprazole in a sample of outpatients with a history of low compliance.

**Methods:**

We conducted a descriptive analysis of a naturalistic sample of 12 patients who switched from oral or monthly LAI aripiprazole to the 960 mg Q2M formulation at ASST FBF Sacco (Milan, Italy). Sociodemographic, clinical, and treatment-related variables were collected, including diagnosis, comorbidities, substance use, hospitalizations, and tolerability.

**Results:**

The sample was evenly distributed by sex (50% female), with a mean age of 29.8 years and a mean duration of untreated psychosis (DUP) of 8.8 years. Half of the patients were diagnosed with schizophrenia spectrum disorders, while 25% had bipolar disorder and 25% personality disorders. Psychiatric comorbidities were present in 50%, and medical comorbidities in 33%. A high prevalence of family psychiatric history (67%) and previous hospitalizations (100%) was observed, with 75% having experienced compulsory admissions (TSO). Most patients (83%) had a history of poor adherence. All had prior exposure to oral and monthly LAI aripiprazole (mainly 400 mg). At baseline with 960 mg Q2M, 66.7% had already received two injections. No adverse events were reported after switching to the bimestral formulation.

**Conclusions:**

This preliminary real-world evidence suggests that switching to aripiprazole LAI 960 mg Q2M is feasible and well-tolerated in a highly complex and non-adherent clinical population. The absence of reported adverse effects, combined with the potential to improve adherence in patients with a severe course of illness, supports further investigation of this treatment option. Larger samples and prospective designs, including clinical scales, are warranted to confirm these encouraging results.

**Disclosure of Interest:**

None Declared

